# Probabilistic Risk Assessment of Combined Exposure to Deoxynivalenol and Emerging *Alternaria* Toxins in Cereal-Based Food Products for Infants and Young Children in China

**DOI:** 10.3390/toxins14080509

**Published:** 2022-07-25

**Authors:** Xiaofeng Ji, Yingping Xiao, Wentao Lyu, Minglu Li, Wen Wang, Biao Tang, Xiaodan Wang, Hua Yang

**Affiliations:** 1 State Key Laboratory for Managing Biotic and Chemical Threats to the Quality and Safety of Agro-Products, Institute of Agro-Product Safety and Nutrition, Zhejiang Academy of Agricultural Sciences, Hangzhou 310021, China; jixiaofeng@zaas.ac.cn (X.J.); ypxiaozaas@hotmail.com (Y.X.); lvwt@zaas.ac.cn (W.L.); wangwen@zaas.ac.cn (W.W.); tb_411@163.com (B.T.); 2 China National Center for Food Safety Risk Assessment, Beijing 100022, China; lml201470699@163.com

**Keywords:** deoxynivalenol, *Alternaria* toxins, combined exposure, cumulative risk assessment

## Abstract

Deoxynivalenol (DON) and emerging *Alternaria* toxins often co-occur in cereal-based products, but the current risk assessment is commonly conducted for only one type of mycotoxin at a time. Compared to adults, infants and young children are more susceptible to mycotoxins through food consumption, especially with cereal-based food products which are the main source of exposure. This study aimed to perform a probabilistic risk assessment of combined exposure to DON and three major *Alternaria* toxins, namely including alternariol monomethyl ether (AME), alternariol (AOH), and tenuazonic acid (TeA) through consumption of cereal-based foods for Chinese infants and young children. A total of 872 cereal-based food products were randomly collected and tested for the occurrence of DON and three major *Alternaria* toxins. The results on mycotoxin occurrence showed the DON, TeA, AOH, and AME was detected in 56.4%, 47.5%, 7.5%, and 5.7% of the samples, respectively. Co-contamination of various mycotoxins was observed in 39.9% of the analyzed samples. A preliminary cumulative risk assessment using the models of hazard index (*HI*) and combined margin of exposure (*MoET*) was performed on DON and *Alternaria* toxins that were present in cereal-based food products for infants and young children in China for the first time. The results showed that only 0.2% and 1.5%, respectively, of individuals exceeded the corresponding reference value for DON and TeA, indicating a low health risk. However, in the case of AME and AOH, the proportion of individuals exceeding the reference value was 24.1% and 33.5%, respectively, indicating the potential health risks. In the cumulative risk assessment of AME and AOH, both *HI* and *MoET* values indicated a more serious risk than that related to individual exposure. Further research is necessary to reduce the uncertainties that are associated with the toxicities of the *Alternaria* toxins and cumulative risk assessment methods.

## 1. Introduction

Mycotoxins are secondary metabolites that are produced by many fungi, including *Aspergillus*, *Fusarium*, *Penicillium*, and *Alternaria* genera under preferable conditions [1]. Mycotoxin contamination can occur frequently in various food products globally, and consuming food products that are contaminated with mycotoxins can lead to a variety of acute or chronic adverse effects including hepatotoxicity, nephrotoxicity, genotoxicity, immunosuppression, endocrine disrupting effects, including carcinogenicity, and teratogenicity [2,3].

Deoxynivalenol (DON), produced by *Fusarium* spp., is a predominantly detected mycotoxin in cereals (e.g., wheat, maize, rye, barley, and oats) and cereal-based food products globally [4,5,6]. DON exposure can cause vomiting, growth retardation, anorexia, reduced nutrient efficiency, and immune dysfunction [7,8]. Although DON is considered as a non-carcinogenic compound, the maximum level (ML) of this toxin has been set up in different countries and international authorities in order to protect human health. The Codex Alimentarius Commission (CAC) sets the ML of DON at 2000 μg/kg in wheat, maize, and barley; the European Union (EU) sets at 1250 μg/kg in μg/kg in unprocessed wheat, maize, and barley; and China sets at 1000 μg/kg in wheat, maize, and wheat flour. The CAC and the European Commission have also set a limit of 200 μg/kg of DON in cereal-based baby foods [9].

Apart from DON, a group of “emerging mycotoxins” that are produced by *Alternaria* spp. found in cereals and cereal-based processed products have also raised concerns in the past few decades [10]. *Alternaria* toxins are often found co-occurring along with DON in cereal-based food products [11,12,13]. Over 70 chemicals belonging to this group have been recognized, among which alternariol monomethyl ether (AME), alternariol (AOH), and tenuazonic acid (TeA) are the most concerning mycotoxins and have been studied previously [14]. *Alternaria* toxins exert multiple potential toxicities, including genotoxicity, cytotoxicity, and endocrine disruption effects [13]. Among *Alternaria* toxins, TeA exerts the highest acute toxicity and can cause hemorrhages in many organs, suppress weight gain, and reduce feed efficiency in animals, whereas AME and AOH were both strongly mutagenic to some bacterial cells [15]. Alarming levels of *Alternaria* toxins have been found in cereal-based baby foods which can cause exposure to an inadequate margin of safety [16]. However, no regulation has been implemented for *Alternaria* toxins in foods globally because of a lack of data on toxicity and exposure, except for the maximum limit of 500 µg/kg set for TeA in sorghum/millet-based baby foods by the Bavarian Health and Food Safety Authority [16].

Infants and young children are more susceptible than adults to the toxic effects of exogenous harmful substances owing to their immature organ development, poor detoxification ability, and high food intake per unit of body weight. They are usually regarded as vulnerable subgroups in food safety risk assessments [17]. In their diet, cereal-based complementary foods make a major contribution to providing energy and nutrition. However, mycotoxin contamination can often occur in these food products, which can be hazardous to their health [18].

Humans are naturally exposed to various chemical hazards with multiple exposure pathways, and different chemical hazards may pose synergized effects on humans. Previous studies mainly focused on single compound exposure assessment for consumers [15,19,20,21], while only a few studies conducted a cumulative risk assessment of mycotoxins [22,23,24]. Different methodologies have been proposed for the assessment of cumulative health risk of exposures to multiple chemicals, which includes hazard index (*HI*), combined margin of exposure (*MoET*), relative potency factor/toxic equivalent factor (RPF/TEF), and reference point index (RPI) [25,26,27,28,29]. While choosing the appropriate models for multiple mycotoxins assessment, their composition in the diet and mode of action (MOA) should be considered. Toxicological parameters of health-based guidance values (HBGVs) and reference dose (RfD), or point of departure (POD) are usually required for the cumulative risk assessment models.

Considering the scarce information on the cumulative risk assessment of DON and *Alternaria* toxins in cereal-based food products, and the fact that cereal-based food products are the main food source in the diet of infants and young children after 6 months, the present study aimed to evaluate the occurrence of DON and *Alternaria* toxins (AME, AOH, and TeA) in cereal-based food products that were marketed in China, and subsequent probabilistic risk assessment of combined exposure to DON and *Alternaria* toxins in cereal-based food products was performed for Chinese infants and young children.

## 2. Results

### 2.1. Occurrence of DON and Alternaria Toxins in Cereal-Based Food Products

The occurrences of DON, AME, AOH, and TeA in cereal-based food products are presented in Table 1, Table 2, Table 3 and Table 4. The data on acylated DON are not provided because of their extremely low positive rates. Among the samples that were analyzed, 70.2% (612/872) of the samples exhibited at least one DON and *Alternaria* toxin, and 39.9% (348/872) of the analyzed samples were co-detected with two or more toxins. As shown in Table 1, DON was detected in 56.4% (492/872) of the samples, with 60.7% (398/656) of the cereal-based complementary foods and 43.5% (94/216) of the common cereals. Among all the cereal-based complementary foods, noodles had the highest positive rate of 91.7% (210/229) followed by biscuits (63.3%, 186/294), whereas only 1.5% (2/133) of the rice flour was positive for DON. Among all the common cereals, maize had the highest positive rate for DON (92.6%, 25/27), followed by wheat flour (74.2%, 46/62). The positive rates of rice and millet were relatively low, 27.6% (16/58) and 10.1% (7/69), respectively. The mean level of DON in all the samples was 26.89 (26.67–27.11) μg/kg with a median of 6.18 μg/kg and a maximum of 912.29 μg/kg (Table 1).

As for AME and AOH, their positive rates in the samples, 5.7% (50/872) for AME and 7.5% (65/872) for AOH, were much lower. Their mean levels in the food samples were also lower compared with the TeA levels; the mean levels of AME and AOH were 0.23 (0.08–0.37) μg/kg (Table 2) and 0.66 (0.52–0.30) μg/kg (Table 3), respectively. Among the different types of foods, the positive rate (37.1%, 23/62) and the mean level 6.36 (6.27–6.46) μg/kg of AOH were considerably higher in the wheat flour samples than in the other foods (Table 3). Similarly, for AME, the wheat flour samples showed a markedly higher positive rate (17.7%, 11/62) and mean level 0.32 μg/kg (0.19–0.44 μg/kg) than other types of food products (Table 2).

For *Alternaria* toxins, TeA showed the highest positive rate and contamination level. As shown in Table 4, its overall positive rate was 47.5% (414/872) and the mean level was 18.26 μg/kg (18.00–18.52 μg/kg). The positive rate of TeA in the cereal-based complementary foods (52.7%, 346/656) was higher than that in the common cereals (31.5%, 68/216), which is a noteworthy result. Among all the food categories, millet had the highest positive rate for TeA (78.3%, 54/69) with a mean level of 73.60 (73.49–73.70) μg/kg. This was followed by noodles for babies with a positive rate and mean level of 89.5% (205/ 229) and 26.81 (26.76–26.87) μg/kg, respectively.

### 2.2. Co-Occurrence of DON and Emerging Alternaria Toxins in Cereal-Based Food Products

**Figure 1:**
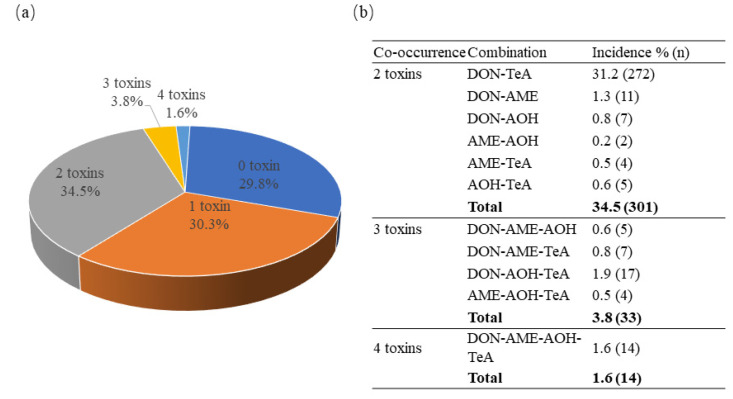
Frequency of co-occurrence of DON, AME, AOH, and TeA (**a**) and combination type of co-occurrence of DON, AME, AOH, and TeA in cereal-based food products (**b**).

Multiple mycotoxins were simultaneously and frequently detected in the cereals as they may be contaminated with multiple fungi (e.g., *Aspergillus*, *Fusarium*, *Penicillium*, and *Alternaria*) [12]. The co-occurrence of DON and *Alternaria* toxins in cereal-based food products is displayed in Figure 1. In this study, about 39.9% (348/872) of the samples were contaminated with two or more toxins, 30.3% (264/872) were contaminated with only one toxin, 34.5% (301/872) with two toxins, 3.8% (33/872) with three toxins, and 1.6% (14/872) with four toxins. Overall, the co-occurrence of DON–TeA (31.2%) was the predominant mycotoxin combination, and the most common combination for the co-occurrence of three toxins was DON-AOH-TeA (Figure 1).

### 2.3. Acute Risk Assessment of DON

A single large dose of mycotoxin can potentially cause acute poisoning. Among the DON and *Alternaria* toxins that were analyzed in this study, only the acute reference dose (ARfD) of DON (8 µg/kg bw) is known, so a probabilistic model based on Monte Carlo simulation was used to determine the acute risk for infants and young children resulting from DON exposure by consuming each type of cereal-based food products. The occurrence data and consumption data of each food product were randomly drawn and multiplied, and the population distribution of DON intake per kg body weight was simulated through 10,000 iterations using the @Risk software version 8.0 (Palisade Corporation, Ithaca, NY, USA). The probability of acute exposure exceeding ARfD of 8 μg/kg bw was calculated in the population [30].

The result of the acute exposure estimate using the probability method showed almost no possibility of exceeding the ARfD of 8 μg/kg bw by consuming the relevant foods, indicating the low risk of acute poisoning in infants and young children.

### 2.4. Chronic Risk Assessment of Individual DON and Alternaria Toxins

Table 5 shows the estimated chronic exposure (daily intake) of DON, AME, AOH, and TeA for each age group of infants and young children under three different scenarios. Regarding the estimated average dietary exposure to DON and *Alternaria* toxins through cereal-based food product consumption, higher exposure was seen in the 1–2 years groups, especially for DON and TeA (up to 0.085 and 0.125 μg/kg bw/day in the MB scenario, respectively). As shown in Table 5, the estimated chronic mean exposure and 95th percentile exposure to DON through the consumption of cereal-based complementary foods and common cereals in infants and young children (0–3) was 0.081 (0.077–0.085) μg/kg bw/day and 0.269 (0.264–0.275) μg/kg bw/day, respectively, with only 0.2% of the individuals exceeding the tolerable daily intake (TDI) (1.0 µg/kg bw/day) of DON in LB, MB, and UB scenarios.

As shown in Table 5, the estimated mean and 95th percentile AME intake from cereal-based food products was about 0.001 (0.0002–0.003) μg/kg bw/day and 0.019 (0.009–0.004) μg/kg bw/day, respectively, across different age groups of infants and young children, with 24.1% of the individuals exceeding the threshold of toxicological concern (TTC) (0.0025 µg/kg bw/day) of AME in LB, MB, and UB scenarios. Similarly, the estimated mean and 95th percentile AOH was 0.003 (0.0002–0.005) μg/kg bw/day and 0.151 (0.148–0.155) μg/kg bw/day, respectively, with 33.5% of the individuals exceeding the TTC (0.0025 µg/kg bw/day) of AOH in three presumed scenarios.

The estimated mean and 95th percentile TeA intake exposure was 0.121 (0.116–0.125) μg/kg bw/day and 3.499 (3.492–3.505) μg/kg bw/day, respectively, in infants and young children (1–3 years), with 1.5% of the individuals exceeding the TDI (1.5 µg/kg bw/day) of TeA in the LB, MB, and UB scenarios (Table 5).

### 2.5. Contribution of Various Cereal-Based Food Products to Total Estimated Dietary Intake of Mycotoxins

**Figure 2:**
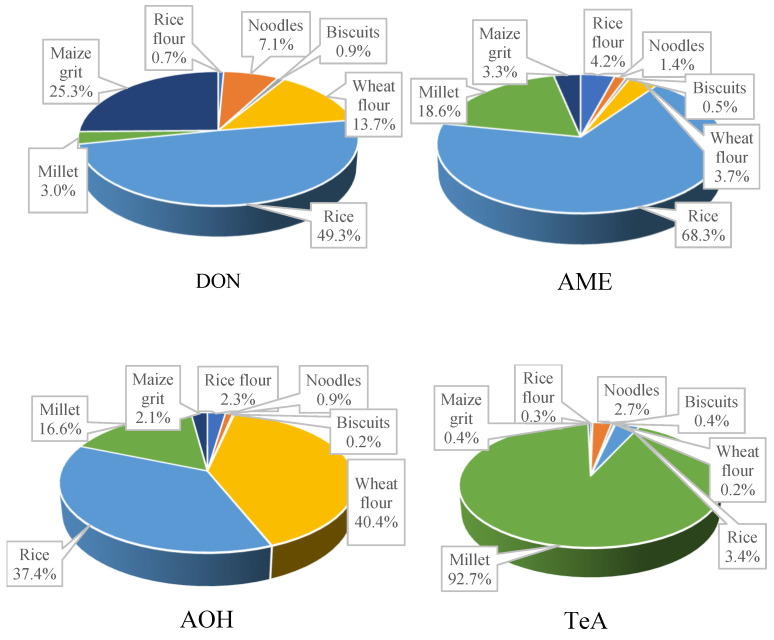
The contribution of each food product to the exposures of DON, AME, AOH, and TeA.

Figure 2 shows the contribution of each cereal-based food commodity to the exposures of DON, AME, AOH, and TeA. Cereal-based complementary foods including rice flour, noodles, and biscuits showed a slight contribution to the total exposure (8.8% to DON, 6.1% to AOH, 3.5% to AME, and 3.4% to TeA, respectively). Among the samples, noodles had the highest contribution to DON (7.1%) and TeA (2.7%) exposure, whereas rice flour had the highest contribution to AME and AOH exposures (4.2% and 2.3%, respectively). Conversely, common cereals contributed to the average of 96.6, 96.5, 93.9, and 91.2% of exposures to TeA, AOH, AME, and DON, respectively, accounting for a predominant percentage of the total exposure. Among common cereals, the mean level of DON in rice (4.84 μg/kg) was much lower than that in wheat flour (52.51 μg/kg) or maize (88.75 μg/kg) (Table 1); however, the contribution of rice was the highest (49.3%). Wheat flour showed the highest contribution to the average AOH exposure (40.5%). Millet showed the highest contribution to the average TeA exposure of by a landslide percentage (92.7%).

**Figure 3:**
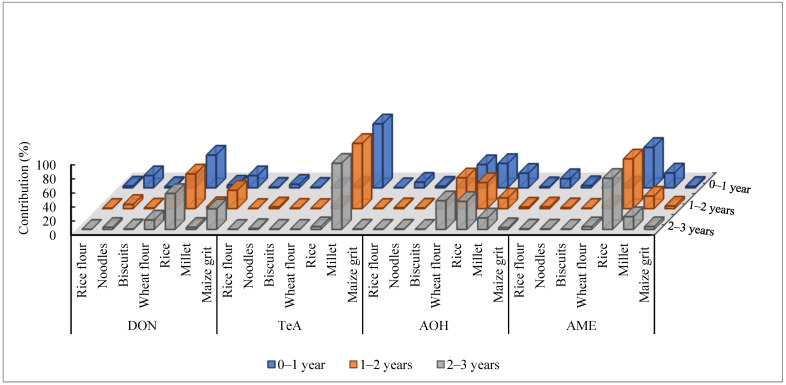
The comparison of contributions (% of estimated daily intake) of cereal-based food products to the DON, AME, AOH, and TeA exposures among Chinese infants and young children of different age groups.

Figure 3 illustrates the comparison of contributions of cereal-based food groups to the DON, AME, AOH, and TeA exposures among Chinese infants and young children of different age groups. Among infants and young children of different age groups, infants at age 0–1 year are exposed more to dietary risk than these in the other two age groups (1–2 years and 2–3 years) because of the consumption of noodles by the latter. Among the common cereals, rice was found to be the major contributor to the dietary risks that are related to DON, AME, and AOH exposures across infants and young children of different age groups, which may be attributed to the consumption of abundant rice by infants and young children in their diet. Maize grit and wheat flour were identified as important contributors to DON and AOH exposures, respectively.

### 2.6. Cumulative Risk Assessment of DON and Emerging Alternaria Toxins

In terms of the combined exposure of mycotoxins, we found that the co-occurrence of DON and the *Alternaria* toxins, especially DON and TeA, is common in cereal-based food products. Due to the considerable possibility of exposure to multiple mycotoxins in consumers, the cumulative risks of the relevant multiple mycotoxins were further evaluated in this study. After taking into account the possible mycotoxin combinations that were displayed by the food analytical data, MoA of different mycotoxins, and the application conditions of different models, the cumulative risks of DON and TeA co-exposure were characterized using an *HI* model, while for AOH–AME co-exposure scenario, both *HI* and *MoET* models were applied.

Table 6 presents the risk characterization using the hazard quotient (*HQ*) and *HI* that were derived from the probabilistic risk assessment of individual and combined exposure to DON, TeA, AME, and AOH in cereal-based food products among infants and young children in China. This study highlighted that all *HQ*s or *HI*s were <1 (0.0–0.60) for DON, TeA, and DON and TeA at the mean, median, 90th, and 95th exposure levels under LB, MB, and UB scenarios, and only 1.5% of the individuals whose Hi values were greater than I under UB, indicating a low health risk to the individual DON and TeA exposure and the combined DON–TeA exposure for infants and young children in China. Among AME, AOH, and AME and AOH, the *Hi*s of AME and AOH were greater than 1 even at P90 under LB scenario, and 13.2% of the individuals showed *HI* values greater than 1, indicating that the cumulative health risks of AME–AOH co-exposure require further attention. In addition, the *Hi*s of the cumulative AME–AOH exposure were higher than the *HQ*s of individual exposure to AME or AOH (Table 6).

Table 7 presents the risk characterization using *MoE* and *MoET* values that were derived from the probabilistic risk assessment of individual and combined exposure to AME and AOH in cereal-based food products for infants and young children in China. The results indicated that all *MoE* and *MoET* values were lower than 10,000 (lower to 5.62 at UB for AME–AOH), and 100% of the individuals exhibited *MoET* values that were lower than 10,000, suggesting serious health risks to infants and young children.

## 3. Discussion

In the present study, 872 cereal-based food products were analyzed for DON and emerging *Alternaria* toxin contamination. DON is a predominant mycotoxin in cereals, and its high incidence and contamination level have been reported in many studies [31,32,33]. As for emerging *Alternaria* toxins, the contamination of AOH and AME in various food categories were frequently detected [34]. The analysis results in this study showed that TeA as the most common detected *Alternaria* toxin, which was consistent with previous studies [20,35].

Ideally, mycotoxin contamination in cereal-based complementary foods for infants and young children is expected to be lesser than that in common cereals; however, the available data in this study indicated that contamination rates in cereal-based complementary foods has a worryingly higher positive rate. For DON, the positive rates in cereal-based complementary foods and common cereals were 60.7% and 43.5%, respectively (Table 1). The high incidence of DON in cereal-based complementary foods has also been reported in Wang et al.’s study [32]. Compared with the survey that has been conducted in other countries, the positive rate of DON in the infant food samples that were collected in this study was much higher (60.7% versus 9.09%) [36]. This is considered to be possibly related to the current status that China has not implemented the DON limit standard for cereal-based complementary foods for infants, and many excipients are added to cereal-based complementary food products during the production process. Moreover, in this study, a higher TeA positive rate was also discovered in the cereal-based complementary foods (52.7%) than in the common cereals (31.5%).

The co-occurrence of DON with emerging *Alternaria* toxins was reported in previous studies [37,38]. Babič et al. (2021) [37] reported that DON and TeA were present in 32% and 26% of cereal grain samples, respectively, whereas AOH, TEN, and AME were detected in fewer than 15% of the cereal grain samples. Juan et al. (2016) [38] reported that 91% of positive wheat samples presented the co-occurrence of more than one mycotoxin, and the detection rates of DON, AOH, and AME were 16%, 31%, and 26%, respectively.

As for the estimation of acute exposure to DON, this study revealed that there was almost no possibility of exceeding the ARfD of 8 μg/kg bw by consuming the relevant foods, indicating the low risk of acute poisoning in infants and young children. On the contrary, a similar study [32] reported that the probability of exceeding the ArfD attributed to 10,000 iterations of eating wheat-based foods was estimated to be 1.9%. in this study, the maximum DON concentration in the cereal-based complementary foods was 912.29 μg/kg, which was close to that which was obtained in a study by Wang et al. (2019) (1198.7 μg/kg), whereas the 95th percentile level (100.12 μg/kg) was much lower than that (600.5 μg/kg) in Wang et al. (2019) [32]. There were two possible reasons for the decrease in high-percentile contamination levels as follows: (1) the climate in different years might affect mycotoxin contamination in the raw materials and processed products. (2) China has begun to consider setting DON maximum limits for cereal-based complementary foods since 2019 and issued a draft for these standards, which forced manufacturers to abandon the use of severely contaminating raw materials.

The estimated mean exposure of DON in this study was similar to that of a previous report that was based on an investigation in Spain [39]. In that report, the estimated daily intake was 0.08 DON μg/kg bw/day through the consumption of cereal-based baby foods. The estimated daily intake for DON from cereal-based baby food in different stages of infancy (4 months–12 months) ranged from 0.25 to 0.36 μg/kg bw/day in Spain [40], representing 25.1% to 36.2% of the TDI (1.0 µg/kg bw/day) of DON that was established by European Food Safety Authority (EFSA), which is obviously higher than that of the results in this study. Those results suggested that DON contamination may not be a serious problem for cereal-based complementary foods and common cereals in China.

Although the estimated exposure to individual AME and AOH seemed low, about a quarter of individuals exceeded the TTC value. Considering the possible serious outcomes of AME and AOH exposure, attention should be paid to them. EFSA also considered them as the two most concerning *Alternaria* toxins after a risk assessment [41]. In addition, Zhao et al. (2015) [15] reported that dietary exposure to AME and AOH in the Chinese general population and different age subgroups exceeded their corresponding TTC value, with the highest exposure detected in children.

Previous published data related to the dietary risk assessment of TeA based on the TTC value are limited. EFSA (2011) [41] reported that the estimated mean TeA intake was less than 0.8 μg/kg bw/day, which was lower than the results that were obtained in this study (0.121 μg/kg bw/day for the mean population distribution). Zhao et al. (2015) [15] reported that the dietary exposure in children was 0.292, 0.115, and 1.537 μg/kg bw/day at mean, 50th percentile, and 97.5th percentile estimation, respectively. The 97.5th percentile dietary exposure was slightly higher than the TTC value (1.5 μg/kg bw/day). Considering the synergistic effects among mycotoxins, the health risks that are associated with TEA cannot be ruled out.

Cereal-based products are an important contributor to mycotoxin exposure globally [42]. Wang et al. (2019) [32] reported that noodles showed the highest contribution to DON (77.8%) exposure in Chinese infants, whereas both rice flour and biscuits showed 11.1% contribution to DON exposure, and these percentages are higher than the values that were obtained in this study. The highest contributions for DON came from grain-based products across all age groups ranging between 65% (in infants) and 94% (in very elderly), and wheat was found to be the largest contributor of DON exposure in Europe (79%) [43]. Arcella, et al. (2016) [44] reported that cereals were one of the main contributors to the *Alternaria* mycotoxin exposure across the European population, which is consistent with the results of this study.

To the best of our knowledge, a cumulative risk assessment was performed on DON and *Alternaria* toxins that were present in cereal-based food products for infants and young children in China for the first time. In this study, the risk characterization of DON and *Alternaria* toxins suggested a low health risk due to the combined DON and TeA exposure to infants and young children in China. However, the combined exposures to AME and AOH indicated a more serious risk than that which was related to individual exposure for infants and young children in China. However, this study has some limitations as follows: the foods that were included in the exposure assessment of the *Alternaria* toxins were cereal-based food products only; however, these mycotoxins also occur in other food categories such as oilseeds, vegetables, and fruits [13,45,46], and the lack of these food sources may underestimate the risks. Due to the inadequate toxicological data on the *Alternaria* toxins and their complex mechanism of toxicity, their adverse toxicological effects have not been fully unraveled; hence, this study adopted a preliminary approach for individual and cumulative risk assessments. In the future, as more studies will be performed to determine the below minimum detectable limits (BMDLs) with more reliable *MoE*s or reference values, a more accurate assessment will be performed to characterize the health risks of exposure to the individual *Alternaria* toxin and the cumulative risks of their combined exposure with other mycotoxins.

Given the uncertainties that are involved in the present study, especially those regarding the toxicities of the *Alternaria* toxins and the cumulative risk assessment methods, further research is needed on the health risks in infants and young children who are getting exposed to single or multiple mycotoxins via cereal-based food products. Although the toxicity data of TeA are limited, its acute toxicity and median lethal dose (LD_50_) in mice seem to be similar to that of DON (46–78 mg/kg bw), which is worth noticing [19]. Moreover, according to the data in this study, the co-occurrence of DON and TeA is the most common combination with an incidence of 31.2%, indicating that the cumulative acute risk of exposure to both substances is of great concern. So far, an ARfD has been established for DON but not for TeA. The assessment of cumulative acute risks in this situation is worth studying. Therefore, in future studies, the health risks of combined exposure to these two mycotoxins should be explored from the perspective of acute toxicity.

Mycotoxins in cereal-based food products mainly come from the raw materials, and mycotoxins are difficult to destroy during processing [47]. Therefore, the key measure to reduce the mycotoxin levels in final food products is the control of mycotoxin contamination in raw cereals, such as wheat and maize in both the pre- and post-harvest phases. Mycotoxin control strategies for cereals should be established according to local specificities and climatic conditions. Several practices have been revealed to be useful, including tillage, cover crop, crop rotation, antifungal mulch treatment, intercropping, and the application of fungal biocontrol agents at the pre-harvest stages as well as the treatment of stored grains with UV light, cleaning, and milling at the post-harvest stages [47]. In addition, setting stricter standards for mycotoxins in cereal-based complementary foods for infants than common foods will help to protect the health of this vulnerable population.

## 4. Conclusions

Considering the prevalence of mycotoxin contamination in cereal-based food products and the vulnerability of infants and young children to these exogenous hazards, we assessed the individual and cumulative health risks of exposure to DON and three major *Alternaria* toxins (AOH, AME, and TeA) in this study. The risk assessment results showed that the health risks that were posed by DON and TeA, and their combined exposure are low despite their considerable prevalence in both cereal-based complementary foods and common cereals. However, the exposures of AOH and AME, individual or combined, from cereal-based food products indicate a potentially unacceptable risk for Chinese infants and young children. Further research is necessary to reduce the uncertainties that are associated with the toxicities of the *Alternaria* toxins and cumulative risk assessment methods.

## 5. Materials and Method

### 5.1. Sample Collection

In this study, we included major cereal-based food products that are consumed by infants and young children to obtain representative exposures. This included common cereals of rice, wheat flour, millet, maize, and cereal-based complementary foods of rice flour, biscuits, and noodles for infants and young children. In 2021, 872 food samples, including 656 pre-packaged complementary foods (294 biscuits, 229 noodles, and 133 rice flour) and 216 common cereals (58 rice, 62 wheat flour, 69 millets, and 27 maize), were collected from the markets (e.g., supermarkets, baby food stores, and online stores) of 18 provinces across China using a stratified random sampling method. Every batch of collected food was thoroughly mixed to obtain a homogeneous sample. All the samples were ground to a fine powder and stored in plastic containers at −20 °C until mycotoxin analysis.

### 5.2. Mycotoxin Analysis

The food samples were analyzed to detect the presence of DON, 3-acetylated DON (3-ADON), 15-acetylated DON (15-ADON), AME, AOH, and TeA based on the method that was reported by Ji et al. (2022) [48]. High-performance liquid chromatography-tandem mass spectrometry (HPLC-MS/MS) was used to evaluate the concentrations of DON and *Alternaria* toxins in the samples. We used an AB Sciex 6500 Qtrap tandem mass spectrometer (Redwood City, CA, USA) coupled with a Shimadzu LC-30AD quaternary pump (Tokyo, Japan), SIL-30AC autosampler, and CTO-20AC column oven. All the reagents were HPLC or analytical grade. The standards of DON, 3-ADON, 15-ADON, AME, AOH, and TeA were purchased from Romer labs (Union City, MO, USA).

The limit of detection (LOD) and limit of quantification (LOQ) was 1.0 µg/kg and 3.0 µg/kg, respectively, for DON, 3-ADON, 15-ADON and TeA, whereas 0.3 µg/kg and 1.0 µg/kg, respectively, for AOH and AME.

### 5.3. Food Consumption Data

Food consumption data of infants and young children were obtained from a nationwide survey of Chinese residents that was conducted by the China National Center for Food Safety Risk Assessment (CFSA) in 2015. In this survey, a multi-stage stratified cluster random sampling method was applied and a validated 24 h dietary recall questionnaire was used to collect the diet information on three discrete days in 15 provinces (autonomous regions and municipalities). The data were recorded through interviewing with the parents or main supporters of the babies face-to-face by well-trained investigators. A total of 19,480 infants and young children were surveyed to obtain data on food consumption. Infants that do not consume cereal-based food products were filtered out, and a total of 16,163 individuals were assessed for exposure in this study.

### 5.4. Exposure Assessment

Exposure assessments for mycotoxins were estimated using a probabilistic model. The target population of this study was infants and young children aged 0–3 years in China. Their mycotoxin exposures from cereal-based food products were estimated based on data about the consumption of the relevant cereal-based food products and the mean levels of the relevant mycotoxins in these cereal-based food products. The probabilistic models were used for exposure assessment, in which the variables were described as distributions instead of point estimates to obtain the overall distribution of the population. The following formula was adopted to calculate the dietary intake (exposure):

(1) $$EXP_{s}=\overset{n}{\underset{i=1}{\sum }}\frac{\left(F_{s,i}\times C_{i}\right)}{BW_{s}}$$

where *EXP_s_* is the daily intake of a certain mycotoxin from all the cereal foods that were involved for a certain individual s (μg/kg bw), *Fs,i* is the consumption of food *i* (kg/d), *Ci* is the mean level of the mycotoxin in food *i* (μg/kg), and *BW* is the body weight of the individual s (kg). When the daily exposures for all 16,163 individuals were obtained, the statistics data, including the mean, P50, P90, and P95 values, were calculated from the distribution of exposures in the population [27].

After calculating the total exposure from all the related foods for each individual, distribution fitting was performed using the software @Risk. Monte Carlo simulations were performed through 10,000 iterations and the best fit function based on the lowest Akaike’s information criterion was applied to obtain the statistics of the mean, median, P90, P95, maximum, and the probability of exceeding the reference values in the lower, medium and upper bound scenarios [30].

### 5.5. Risk Characterization

Risk characterization was performed by comparing the outputs of exposure with the reference dose values. As for DON, a traditional mycotoxin, widely accepted acute and chronic HBGVs, namely ARfD and TDI, have been established by the Joint FAO/WHO Expert Committee on Food Additives (JECFA) and EFSA based on a comprehensive review of its acute and chronic toxicity data. ARfD and TDI are 8 μg/kg bw/d and 1 μg/kg bw/d, respectively [43,49]. However, for *Alternaria* toxins, their toxicity data are not sufficient yet. Therefore, the TTC value was used as a reference dose for AME, AOH, and TeA. The TTC value of 0.0025 μg/kg bw/day was adopted for AME and AOH, and the TTC value of 1.5 μg /kg bw/day was adopted for TeA [50].

Different methodologies are available for cumulative risk characterization. Based on available toxicological data and application conditions, the *HI* and *MoET* models were used in this study to assess the cumulative risks. The *HI* model is based on the concentration addition concept and provides a measure of the total risk based on the sum of the individual risks of each component. It applies to chemical mixtures in which the components have the same mode of action (MoA) or act on the same target (at the cell, tissue, or organ level) [27]. As previously mentioned, both DON and TeA may cause vomiting, gastroenteritis, suppression in weight gain, and a reduction in feed efficiency. They were considered to be suitable for the method of *HI*. The same is true for AOH and AME, both of which exert genotoxicity in vivo through similar MoA and were also applied to the *HI* model.

The hazard index (*HI*) was calculated as the sum of the hazard quotient (*HQ*) of a group of substances under study. *HQ* is the ratio of the outputs of exposure to the reference dose values. The calculation formula is as follows:

(2) $$HQ_{ij}=\frac{Exp_{ij}}{RfD_{j}}\;HI_{i}=\overset{n}{\underset{j=1}{\sum }}HQ_{ij}=\overset{n}{\underset{j=1}{\sum }}\frac{Exp_{ij}}{RfD_{j}}$$

where *HQ_ij_* is the hazard quotient of the individual *i* that is exposed to substance *j*; *Exp_ij_* is the daily intake of a substance *j* by the individual *i* (μg/kg bw/day), and *RfD_j_* is the reference dose of the substance *j* (μg /kg bw/day).

*HI_i_* is the hazard index of the combined exposure level to the mycotoxin mixture by individual *I*, and *n* is the total number of substances under evaluation in the mixture. *HI* > 1 indicates a non-tolerable exposure to the mycotoxin mixture.

Alternatively, the *MoET* model is suitable for the assessment of the cumulative risk of genotoxic or carcinogenic chemicals. As AOH and AME can exert potential genotoxicity in vivo, their cumulative health risks were also characterized using the index of *MoET*, which is the reciprocal of the sum of the reciprocals of the margin of exposure (*MoE*) for each mycotoxin that is involved, as expressed by the following formula:

(3) $$MoE_{ij}=\frac{PoD_{j}}{Exp_{ij}}\;MoET_{i}=\frac{1}{\sum_{i=1}^{n}\frac{1}{MoE_{ij}}}$$

where *MoE_ij_* is the *MoE* of the individual *i* that is exposed to substance *j*; *PoD_j_* is the point of departure for the substance *j* (μg/kg), Exp*_ij_* is the daily exposure of the individual *i* to the substance *j* (μg /kg bw/day), *MoET_i_* is the total *MoE* of the individual *i* that is exposed to the mixture that is composed of n mycotoxins. A higher *MoE* indicates a lower risk. Although no established criteria have been set for the magnitude of an acceptable *MoE*, an *MoE* or *MoET* of 10,000 for genotoxic and carcinogenic chemicals that was derived from animal experiments was considered of low concern for public health by EFSA [27]. Hence, in this study, an *MoE* or *MoET* below 10,000 was regarded as unacceptable.

As the existing toxicological data are insufficient for *Alternaria* toxins, very few studies were available that could derive a BMDL for AOH and AME. Therefore, no widely acknowledged BMDL has been established yet, and only a 28-day in vivo study in Sprague-Dawley rats showing a BMDL_10_ value of 0.0336 μg/kg bw/day for AME was reported [51]. Given the similar structure and MoA of AOH and AME, the same BMDL_10_ value was provisionally assumed for AOH and AME for the cumulative risk assessment in the present study.

In this study, three different scenarios were included to facilitate the exposure assessment in relation to the treatment of left-censored data (testing results below LOD). During data analysis, the left-censored data were substituted for 0, half of LOD, and LOD, which was regarded as the lower bound (LB), middle bound (MB), and upper bound (UB), respectively [52].

## Figures and Tables

**Table 1:** Occurrence of DON in cereal-based food products (µg/kg).

Food Category	*n*	Positive (%)	Mean Occurrence MB (LB-UB) (µg/kg)	Median of Occurrence MB (LB-UB) (µg/kg)	P90 of Occurrence MB (LB-UB) (µg/kg)	P95 of Occurrence MB (LB-UB) (µg/kg)	Maximum (µg/kg)
CCF	656	60.7	26.53 (26.34–26.73)	7.94	69.50	95.99	912.29
Rice flour	133	1.50	1.12 (0.63–1.62)	0.50 (0.00–1.00)	0.50 (0.00–1.00)	0.50 (0.00-1.00)	65.52
Noodles	229	91.7	48.09 (48.05–48.13)	29.67	98.14	133.23	912.29
Biscuits	294	63.3	21.24 (21.06–21.42)	7.62	52.95	81.88	282.22
Common cereals	216	43.5	27.97 (27.69–28.25)	0.50 (0.00–1.00)	76.6	146.08	622.40
Rice	58	27.6	4.84 (4.48–5.20)	0.50 (0.00–1.00)	19.14	25.40	34.50
Wheat flour	62	74.2	52.51(52.38–52.64)	21.60	102.00	158.28	622.40
Millet	69	10.1	1.58 (1.13–2.03)	0.50 (0.00–1.00)	1.21 (0.81–1.61)	8.77	23.19
Maize	27	92.6	88.75 (88.71–88.79)	40.00	250.74	328.61	391.20
Total	872	56.4	26.89 (26.67–27.11)	6.18	71.34	100.12	912.29

CCF: Cereal-based complementary food. *n* = number of samples. Mean = arithmetic mean; median = 50th percentile; P90 = 90th percentile; P95 = 95th percentile. MB (LB-UB): middle bound (lower bound-upper bound), LB results below the LOD were replaced with 0; MB: results below the LOD were replaced with LOD/2, UB: results below the LOD were replaced with the value of LOD.

**Table 2:** Occurrence of AME in cereal-based food products (µg/kg).

Food Category	*n*	Positive (%)	Mean Occurrence MB (LB–UB) (µg/kg)	Median of Occurrence MB (LB–UB) (µg/kg)	P90 of Occurrence MB (LB–UB) (µg/kg)	P95 of Occurrence MB (LB–UB) (µg/kg)	Maximum (µg/kg)
CCF	656	5.0	0.22 (0.08–0.36)	0.15 (0.00–0.30)	0.15 (0.00–0.30)	0.35 (0.24–0.47)	7.15
Rice flour	133	0.0	0.15 (0.00–0.30)	0.15 (0.00–0.30)	0.15 (0.00–0.30)	0.15 (0.00–0.30)	0.30
Noodles	229	4.8	0.21 (0.07–0.35)	0.15 (0.00–0.30)	0.15 (0.00–0.30)	0.15 (0.00–0.30)	3.10
Biscuits	294	7.5	0.26 (0.12–0.40)	0.15 (0.00–0.30)	0.15 (0.00–0.30)	1.14	7.15
Common cereals	216	7.9	0.23 (0.10–0.37)	0.15 (0.00–0.30)	0.15 (0.00–0.30)	0.69	3.20
Rice	58	0.0	0.15 (0.00–0.30)	0.15 (0.00–0.30)	0.15 (0.00–0.30)	0.15 (0.00–0.30)	0.30
Wheat flour	62	17.7	0.32 (0.19–0.44)	0.15 (0.00–0.30)	0.59	1.21	3.20
Millet	69	4.4	0.22 (0.08–0.37)	0.15 (0.00–0.30)	0.15 (0.00–0.30)	0.15 (0.00–0.30)	3.20
Maize	27	11.1	0.26 (0.13–0.39)	0.15 (0.00–0.30)	0.49 (0.40–0.58)	1.14	1.20
Total	872	5.7	0.23 (0.08–0.37)	0.15 (0.00–0.30)	0.15 (0.00–0.30)	0.72	7.15

CCF: Cereal-based complementary food. *n* = number of samples. Mean = arithmetic mean; median = 50th percentile; P90 = 90th percentile; P95 = 95th percentile. MB (LB-UB): middle bound (lower bound-upper bound), LB results below the LOD were replaced with 0; MB: results below the LOD were replaced with LOD/2, UB: results below the LOD were replaced with the value of LOD.

**Table 3:** Occurrence of AOH in cereal-based food products (µg/kg).

Food Category	*n*	Positive (%)	Mean Occurrence MB (LB–UB) (µg/kg)	Median of Occurrence MB (LB–UB) (µg/kg)	P90 of Occurrence MB (LB–UB) (µg/kg)	P95 of Occurrence MB (LB–UB) (µg/kg)	Maximum (µg/kg)
CCF	656	4.1	0.21 (0.07–0.36)	0.15 (0.00–0.30)	0.15 (0.00–0.30)	0.15 (0.00–0.30)	3.17
Rice flour	133	0	0.15 (0.00–0.30)	0.15 (0.00–0.30)	0.15 (0.00–0.30)	0.15 (0.00–0.30)	0.30
Noodles	229	7.9	0.26 (0.12–0.40)	0.15 (0.00–0.30)	0.15 (0.00–0.30)	1.27	3.17
Biscuits	294	3.1	0.21 (0.06–0.35)	0.15 (0.00–0.30)	0.15 (0.00–0.30)	0.15 (0.00–0.30)	3.07
Common cereals	216	17.6	2.02 (1.89–2.14)	0.15 (0.00–0.30)	1.20	19.93	45.20
Rice	58	0	0.15 (0.00–0.30)	0.15 (0.00–0.30)	0.15 (0.00–0.30)	0.15 (0.00–0.30)	0.30
Wheat flour	62	37.1	6.36 (6.27–6.46)	0.15 (0.00–0.30)	27.78	30.13	45.20
Millet	69	15.9	0.36 (0.23–0.48)	0.15 (0.00–0.30)	1.05	1.23	3.46
Maize	27	14.8	0.30 (0.17–0.43)	0.15 (0.00–0.30)	1.00	1.07	1.60
Total	872	7.5	0.66 (0.52–0.80)	0.15 (0.00–0.30)	0.15 (0.00–0.30)	1.14	45.20

CCF: Cereal-based complementary food. *n* = number of samples. Mean = arithmetic mean; median = 50th percentile; P90 = 90th percentile; P95 = 95th percentile. MB (LB-UB): middle bound (lower bound-upper bound), LB results below the LOD were replaced with 0; MB: results below the LOD were replaced with LOD/2, UB: results below the LOD were replaced with the value of LOD.

**Table 4:** Occurrence of TeA in cereal-based food products (µg/kg).

Food Category	*n*	Positive (%)	Mean Occurrence MB (LB–UB) (µg/kg)	Median of Occurrence MB (LB–UB) (µg/kg)	P90 of Occurrence MB (LB–UB) (µg/kg)	P95 of Occurrence MB (LB–UB) (µg/kg)	Maximum (µg/kg)
CCF	656	52.7	16.32 (16.08–16.55)	5.08	43.38	58.62	166.36
Rice flour	133	0.8	0.69 (0.19–1.19)	0.50 (0.00–1.00)	0.50 (0.00–1.00)	0.50 (0.00–1.00)	25.78
Noodles	229	89.5	26.81 (26.76–26.87)	21.50	51.45	83.84	164.20
Biscuits	294	47.6	15.21 (14.94–15.47)	0.50 (0.00–1.00)	43.88	58.19	166.36
Common cereals	216	31.5	24.17 (23.83–24.52)	0.50 (0.00–1.00)	52.63	121.50	788.29
Rice	58	0	0.50 (0.00–1.00)	0.50 (0.00–1.00)	0.50 (0.00–1.00)	0.50 (0.00–1.00)	1.00
Wheat flour	62	9.7	0.99 (0.54–1.45)	0.50 (0.00–1.00)	0.50 (0.00–1.00)	4.59	8.30
Millet	69	78.3	73.60 (73.49–73.70)	30.37	140.98	250.90	788.29
Maize	27	29.6	1.96 (1.61–2.31)	0.50 (0.00–1.00)	5.36	6.65	11.00
Total	872	47.5	18.26 (18.00–18.52)	0.50 (0.00–1.00)	44.12	68.65	788.29

CCF: Cereal-based complementary food. *n* = number of samples. Mean = arithmetic mean; median = 50th percentile; P90 = 90th percentile; P95 = 95th percentile. MB (LB-UB): middle bound (lower bound-upper bound), LB results below the LOD were replaced with 0; MB: results below the LOD were replaced with LOD/2, UB: results below the LOD were replaced with the value of LOD.

**Table 5:** Chronic exposures to DON, AME, AOH, and TeA in cereal-based food products for infants and young children in China (µg/kg bw/day).

Mycotoxin	Age Group	Exposure MB (LB–UB)					TDI or TTC	Exceeding RfD^*^ (%)
		Mean	Median	P90	P95	Maximum		
DON	0–1year	0.060 (0.057–0.064)	0.028 (0.025–0.032)	0.015 (0.142–0.153)	0.228 (0.218–0.235)	1.186 (1.853–1.870)	1.0	0.2
	1–2 years	0.090 (0.086–0.095)	0.053 (0.048–0.057)	0.187 (0.180–0.194)	0.296 (0.291–0.303)	6.476 (6.471–6.479)		0.3 (0.3–1.1)
	2–3 years	0.086 (0.082–0.090)	0.050 (0.046–0.054)	0.177 (0.170–0.184)	0.282 (0.276–0.285)	3.335 (3.328–3.341)		0.2
	Total	0.081 (0.077–0.085)	0.046 (0.042–0.049)	0.173 (0.167–0.181)	0.269 (0.264–0.275)	6.476 (6.471–6.479)		0.2
AME	0–1year	0.001 (0.0002–0.0002)	0.001 (0–0.002)	0.003.32 (0.55–6.29)	0.004 (0.0009–0.008)	0.015 (0.003–0.030)	0.0025	17.3 (0–40.4)
	1–2 years	0.001 (0.0002–0.004)	0.002 (0–0.003)	0.003.91 (0.67–7.42)	0.004.82 (0.001–0.009)	0.019 (0.009–0.036)		27.2 (0–57.6)
	2–3 years	0.001 (0.0002–003)	0.002 (0–0.002)	0.003.64 (0.63–6.94)	0.004.48 (0.001–0.009)	0.012 (0.005–0.024)		25.7 (0–56.9)
	Total	0.001 (0.0002–003)	0.001 (0–0.002)	0.003.69 (0.62–7.02)	0.004.63 (0.001–0.001)	0.019 (0.009–0.004)		24.1 (0–52.5)
AOH	0–1years	0.003 (0.001–0.004)	0.001 (0–0.002)	0.005 (1.97–8.07)	0.008 (0.005–0.012)	0.121 (0.119–0.123)	0.0025	26 (12.5–45.3)
	1–2 years	0.004 (0.002–0.005)	0.002 (0–0.003)	0.006 (3.20–10.07)	0.012 (0.010–0.016)	0.151 (0.148–0.155)		37.4 (29.3–62.1)
	2–3 years	0.003 (0.002–0.005)	0.002 (0–0.003)	0.006 (2.98–9.18)	0.011 (0.009–0.012)	0.141 (0.138–0.143)		35.8 (24.7–61.8)
	Total	0.003 (0.002–0.005)	0.002 (0–0.003)	0.006 (2.71–9.25)	0.010 (0.008–0.014)	0.151 (0.148–0.155)		33.5 (23.5–56.8)
TeA	0–1 year	0.117 (0.113–0.120)	0.008 (0.0007–0.015)	0.357 (0.354–0.359)	0.620 (0.617–0.622)	3.205 (3.201–3.210)	1.5	1.5 (0–1.8)
	1–2 years	0.125 (0.119–0.129)	0.009(0.0001–0.019)	0.393 (0.389–0.395)	0.642 (0.639–0.649)	3.499 (3.492–3.505)		1.2 (0–1.5)
	2–3 years	0.120 (0.115–0.125)	0.008 (0–0.016)	0.394 (0.391–0.398)	0.657 (0.653–0.660)	2.459 (2.449–2.468)		3.1 (0–3.8)
	Total	0.121 (0.116–0.125)	0.009 (0.0032–0.017)	0.384 (0.380–0.387)	0.642 (0.639–0.645)	3.499 (3.492–3.505)		1.5 (0–3.4)

bw: body weight; mean, median, P90, P95, and maximum refer to the distribution of the population at the age of 1–3 years. TDI: tolerable daily intake, µg/kg bw/day. TTC: threshold of toxicological concern, µg/kg bw/day. RfD^*^: reference dose, µg/kg bw/day.

**Table 6:** Risk characterization using *HQ* and *HI* that were derived from the probabilistic risk assessment of individual and combined exposure to DON, TeA, AME, and AOH in cereal-based food products for infants and young children in China.

		*HQ*				*HI*	
		DON	TeA	AME	AOH	DON-TeA	AME-AOH
LB	Mean	0.08	0.08	0.08	0.69	0.15	0.77
	Median	0.04	0.0	0.0	0.0	0.07	0.0
	P90	0.17	0.25	0.25	1.09	0.39	1.44
	P95	0.26	0.43	0.38	3.3	0.58	3.58
	Maximum	6.47	2.33	3.69	59.52	6.59	61.35
	*HQ* or *HI* ≥ 1 *n* (%)	43 (0.3)	104 (0.6)	61 (0.4)	1727 (10.7)	220 (1.4)	2130 (13.2)
MB	Mean	0.08	0.08	0.72	1.32	0.16	2.04
	Median	0.05	0.01	0.57	0.68	0.08	1.29
	P90	0.17	0.26	1.48	2.25	0.40	3.89
	P95	0.27	0.43	1.85	4.18	0.59	5.94
	Maximum	6.48	2.33	7.76	60.42	6.61	65.39
	*HQ* or *HI* ≥ 1 *n* (%)	45 (0.3)	104 (0.6)	3887 (24.1)	5357 (33.1)	229 (1.4)	9871 (61.1)
	Mean	0.08	0.08	1.37	1.95	0.17	3.32
UB	Median	0.05	0.01	1.09	1.24	0.09	2.39
	P90	0.18	0.26	2.81	3.70	0.41	6.67
	P95	0.28	0.43	3.53	5.60	0.60	8.87
	Maximum	6.48	2.34	14.6	62.37	6.63	70.53
	*HQ* or *HI* ≥ 1 *n* (%)	46 (0.3)	107 (0.7)	8667 (53.6)	9559 (59.1)	239 (1.5)	12,869 (79.6)

*HQ*: hazard quotient. *HI*: hazard index; *HI* is calculated as the sum of the respective *HQ*. *n*: number of people.

**Table 7:** Risk characterization using *MoE* and *MoET* that were derived from the probabilistic risk assessment of individual and combined exposure to AME and AOH in cereal-based food products for infants and young children in China.

		*MoE*	*MoE*	*MoET*
		AME	AOH	AME-AOH
LB	Mean	93.31	45.82	27.28
	Median	50.87	17.27	12.4
	P90	205.35	97.83	62.99
	P95	301.08	163.34	100
	Maximum	3801.81	7779.81	2553.82
	*MoE* or *MoET* < 10,000 *n* (%)	16,163 (100)	16,163 (100)	16,163 (100)
MB	Mean	49.20	43.23	22.48
	Median	23.45	19.90	10.45
	P90	97.43	85.53	44.78
	P95	162.62	144.53	74.75
	Maximum	3326.4	3326.4	1663.2
	*MoE* or *MoET* < 10,000 *n* (%)	16,163 (100)	16,163 (100)	16,163 (100)
UB	Mean	26.18	23.51	12.14
	Median	12.38	10.88	5.62
	P90	52.14	47.29	24.13
	P95	89.04	79.56	40.88
	Maximum	1663.2	1663.2	831.6
	*MoE* or *MoET* < 10,000 *n* (%)	16,163 (100)	16,163 (100)	16,163 (100)

*n*: number of people *MoE*: the margin of exposure. *MoET*: the combined margin of exposure; *MoET* is calculated as the reciprocal of the sum of the reciprocals of the individual *MoE*.

## Data Availability

The data presented in this study are available in this article.
